# Thymol mitigates lipopolysaccharide-induced endometritis by regulating the TLR4- and ROS-mediated NF-κB signaling pathways

**DOI:** 10.18632/oncotarget.15373

**Published:** 2017-02-16

**Authors:** Haichong Wu, Kangfeng Jiang, Nannan Yin, Xiaofei Ma, Gan Zhao, Changwei Qiu, Ganzhen Deng

**Affiliations:** ^1^ Department of Clinical Veterinary Medicine, College of Veterinary Medicine, Huazhong Agricultural University, Wuhan 430070, People's Republic of China

**Keywords:** thymol, reactive oxygen species, TLR4, nuclear factor-κB, inflammation

## Abstract

The purpose of this study was to investigate the effects of thymol on lipopolysaccharide (LPS)-induced inflammatory responses and to clarify the potential mechanism of these effects. LPS-induced mouse endometritis was used to confirm the anti-inflammatory action of thymol *in vivo*. RAW264.7 cells were used to examine the molecular mechanism and targets of thymol *in vitro*. *In vivo*, thymol markedly alleviated LPS-induced pathological injury, myeloperoxidase (MPO) activity, and the production of tumor necrosis factor-α (TNF-α) and interleukin-1β (IL-1β) in mice. Further studies were performed to examine the expression of the Toll-like receptor 4 (TLR4) -mediated nuclear factor-κB (NF-κB) pathway. These results showed that the expression of the TLR4-mediated NF-κB pathway was inhibited by thymol treatment. *In vitro*, we observed that thymol dose-dependently inhibited the expression of TNF-α, IL-1β, inducible nitric oxide synthase (iNOS), and cyclooxygenase-2 (COX-2) in LPS-stimulated RAW264.7 cells. Moreover, the results obtained from immunofluorescence assays also indicated that thymol dose-dependently suppressed LPS-induced reactive oxygen species (ROS) production. Silencing of TLR4 inhibited NF-κB pathway activation. Furthermore, H_2_O_2_ treatment increased the phosphorylation of p65 and IκBα, which were decreased when treated with *N*-acetyl cysteine or thymol. In conclusion, the anti-inflammatory effects of thymol are associated with activation of the TLR4 or ROS signaling pathways, contributing to NF-κB activation, thereby alleviating LPS-induced oxidative and inflammatory responses.

## INTRODUCTION

Endometritis, defined as inflammation of the uterus, is a prevalent disease in high-producing dairy cows and consequently results in economic losses [1, 2]. The endometrium is the first line of defense against invading pathogenic microorganisms, which cause histological lesions and inflammation of the endometrium [3]. *E. coli* is the major pathogenic bacteria in uterine infection, which produces the endotoxin lipopolysaccharide (LPS) [4]. LPS, an essential constituent of the outer membrane of Gram-negative bacteria, is one of the most efficient stimulators in the immune system [5]. It has been reported that LPS can induce TLR4 signaling pathway activation, which subsequently leads to an overexpression of inflammatory cytokines [6]. TLRs are important factors in the innate immune response and quickly activate multiple pathways upon recognition of microbial pathogens [7]. Many studies have demonstrated that when the expression of TLR4 is stimulated by LPS, it induces nuclear factor (NF)-κB pathway activation [8, 9]. NF-κB is important for inflammatory responses and mediating the secretion of cytokines, such as tumor necrosis factor-α (TNF-α), interleukin-1β (IL-1β), and interleukin-6 (IL-6), to aggravate inflammatory damage [10, 11].

Previous studies have shown that reactive oxygen species (ROS) serve as second messengers and participate in a large number of signal transduction pathways, including those of NF-κB [12, 13]. Pro-inflammatory cytokines, such as IL-1β and TNF-α, promote ROS generation and deteriorate the inflammatory response [14]. Moreover, COX-2 and iNOS also play a predominant role in LPS-stimulated inflammation [15]. Jung et al. have reported suppression of LPS-induced ROS excessive secretion via obstruction of the expression of iNOS and COX-2 [16]. Therefore, this pathway may be used as the theoretical basis for a potential drug for the treatment of inflammatory diseases.

**Figure 1:**
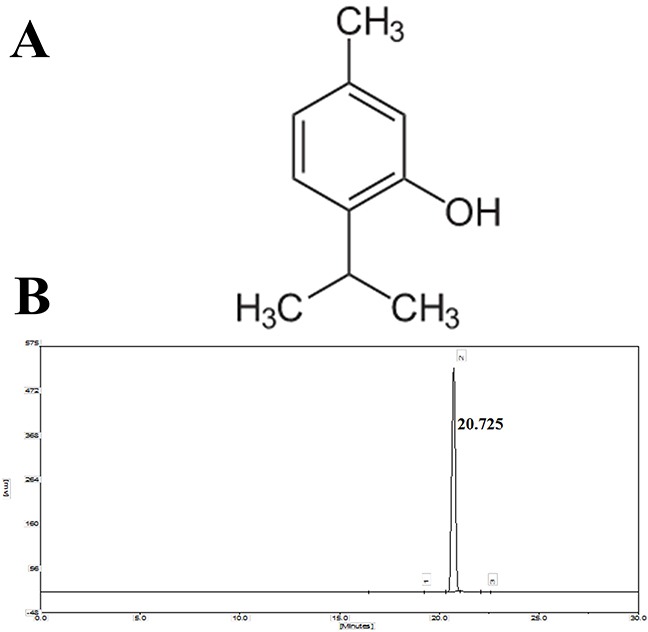
A. Chemical structure of thymol, B. HPLC chromatogram of thymol

Thymol (2-isopropyl-5-methylphenol, Figure 1A), a phenol derivative, is a main component of the essential oil of thyme and is widely used as a fragrance agent in a variety of household products [17]. It has wide-ranging pharmacological actions, such as anti-diabetic, anti-inflammatory, and antioxidant effects [18–20]. Although some studies on the antioxidant effects of thymol have been reported [21, 22], no research has shown the effect of thymol on LPS-induced endometritis in mice. In addition, macrophages stimulate and secrete cytokines during the inflammation cycle and then regulate the innate inflammatory system [23]. Thus, in the present study, mice with LPS-induced endometritis *in vivo* and RAW264.7 macrophages *in vitro* were used to examine the anti-inflammatory molecular mechanism of thymol.

## RESULTS

### *In vivo* study: Effect of thymol on LPS-induced histopathological changes

**Figure 2:**
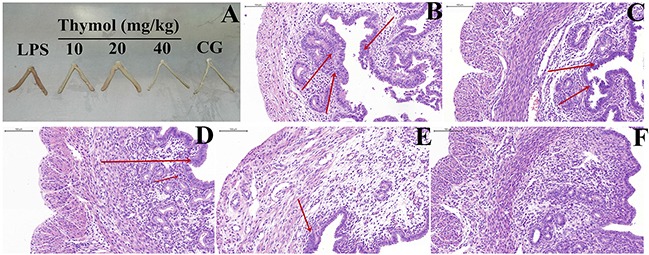
Effects of thymol on LPS-stimulated uterine injury **A**. Morphology of the uterus. **B**. LPS group, **C, D, E**. LPS + Thymol (10, 20, and 40 mg/kg, respectively) treatment groups, **F**. Control group. CG indicates the control group. LPS indicates the LPS-stimulated group. The red arrow indicates the tissue lesion area. Data represent the mean ± S.E.M. of three independent experiments. ^#^p<0.01 *vs*. Control group. *p<0.05 *vs*. LPS group. **p<0.01 *vs*. LPS group.

The severity of LPS-inducedendometritis was determined by histological analysis using H&E staining. Uterus morphology was observed in each group (Figure 2A). Compared with the control group (Figure 2F), the LPS group showed a more severely damaged uterus, with extensive inflammatory cell infiltration (Figure 2B). However, the inflammatory cell infiltration was reduced, and the structure of the uterus was comparatively complete in the thymol groups (Figure 2C-E).

### Effect of thymol on MPO activity and the production of inflammatory mediators

**Figure 3:**
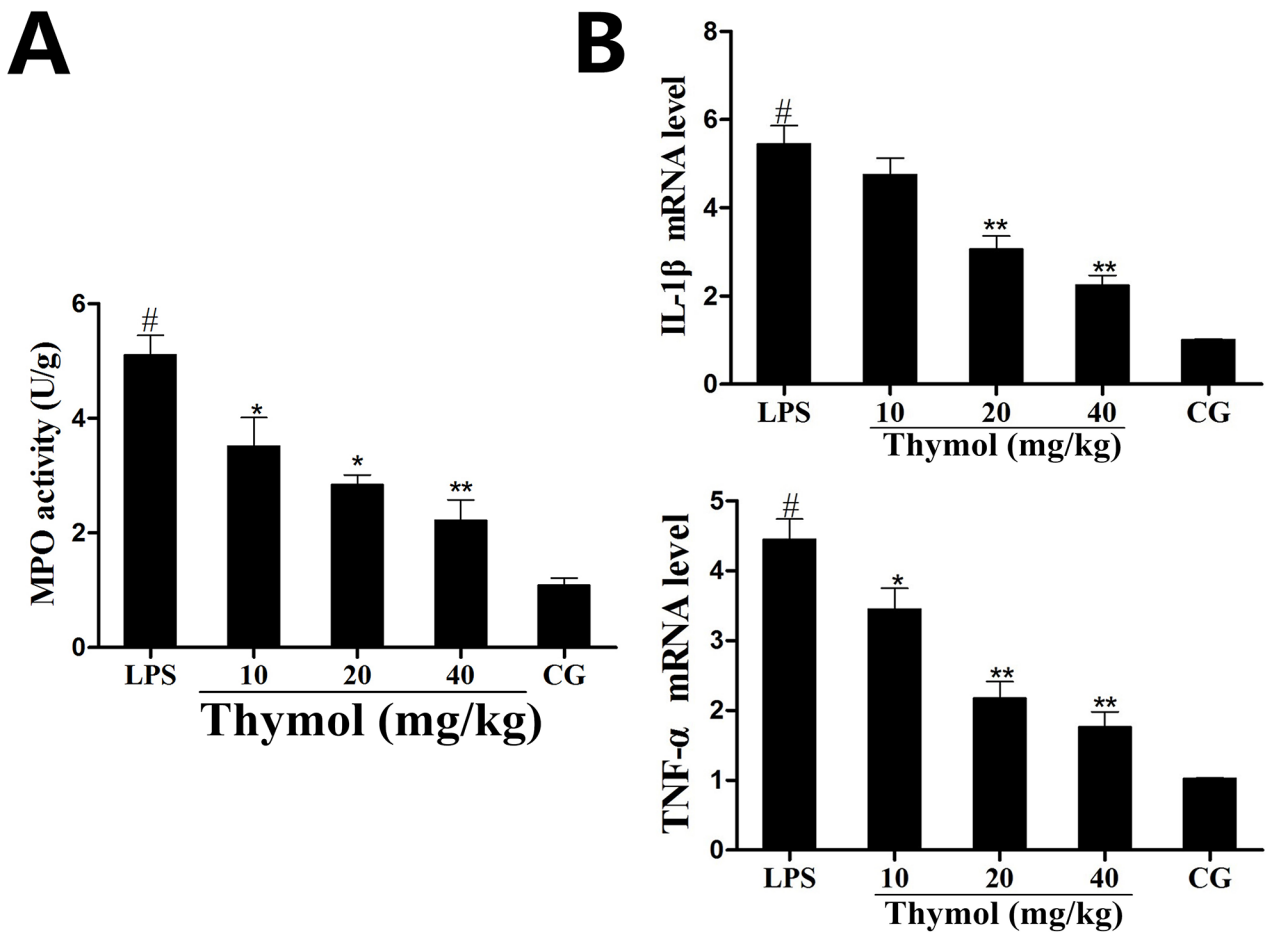
Effects of thymol on MPO activity and cytokine expression **A**. MPO activity. **B**. Expression of TNF-α and IL-1β mRNA in tissues. *GAPDH* serves as the control. CG indicates the control group. LPS indicates the LPS-stimulated group. Data represent the mean ± S.E.M. of three independent experiments. ^#^p<0.01 *vs*. Control group. *p<0.05 *vs*. LPS group, **p<0.01 *vs*. LPS group.

MPO, an early marker in the prediction of inflammatory diseases, reflects the level of inflammation and oxidative stress [24]. As shown in Figure 3A, after administration with LPS, the MPO activity was greatly increased in the LPS group. However, the MPO activity was reduced in a dose-dependent manner by pretreatment with thymol at concentrations of 10, 20 and 40 mg/kg.

The expression of the pro-inflammatory mediators IL-1β and TNF-α were detected using a qPCR assay of the LPS-induced endometritis treatment. The results showed that the levels of TNF-α and IL-1β were significantly increased after LPS treatment, whereas treatment with thymol dose-dependently reduced the expression of these cytokines (Figure 3B).

### Effect of thymol on the expression of TLR4

**Figure 4:**
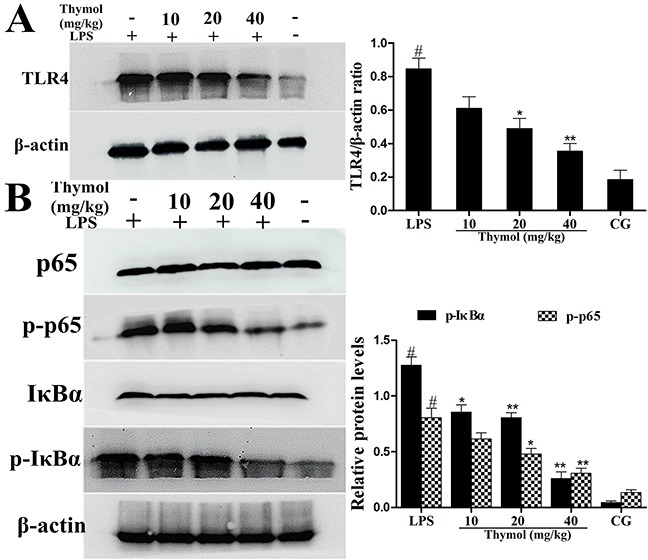
Effects of thymol on TLR4 expression and NF-κB pathway activation **A**. TLR4 protein expression levels in uterine tissues. **B**. Expression of the p65 and IκBα proteins in uterine tissues. Phosphorylation of p65 and IκBα was analyzed using phospho-specific antibodies. β-actin served as an internal control. CG indicates the control group. LPS indicates the LPS-stimulated group. Data represent the mean ± S.E.M. of three independent experiments. ^#^p<0.01 *vs*. Control group. *p<0.05 *vs*. LPS group, **p<0.01 *vs*. LPS group.

To detect whether thymol could inhibit the inflammatory response by suppressing TLR4 expression, western blotting was performed to determine TLR4 expression. As shown in Figure 4A, these results showed that the expression of TLR4 was inhibited by thymol in LPS-induced endometritis.

### Effect of thymol on NF-κB pathway activation

**Figure 5:**
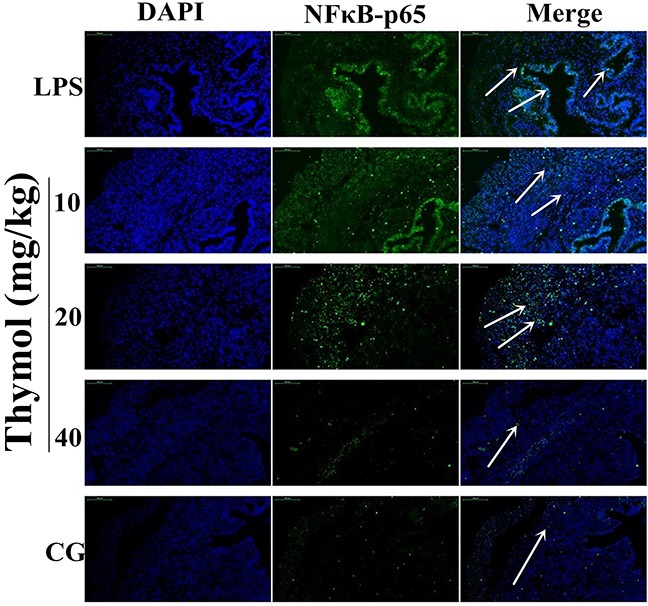
Effects of thymol on NF-κB p65 translocation into the nucleus Paraffin-embedded uterine tissue sections were used to detect p65 translocation to the nucleus using immunofluorescence. **A**. Control group, **B**. LPS group, **C, D, E**. LPS + Thymol (10, 20, and 40 mg/kg, respectively) treatment groups. The white arrow indicates the translocation of p65. Data represent the mean ± S.E.M. of three independent experiments. ^#^p<0.01 *vs*. Control group. *p<0.05 *vs*. LPS group, **p<0.01 *vs*. LPS group.

It is well known that the NF-κB signaling pathway plays a vital role in the production of inflammatory cytokines. To examine whether the suppression of the inflammatory response by thymol occurs via inhibition of the NF-κB pathway activation, western blotting was performed. The results indicated that thymol markedly inhibited the phosphorylation of p65 and IκBα proteins (Figure 4B). To confirm these results, immunofluorescence assays were performed to determine if p65 translocated to the nucleus. As shown in Figure 5, the expression of p65 was significantly decreased in the nucleus with the administration of thymol.

### *In vitro* study: Effect of thymol on cell viability

**Figure 6:**
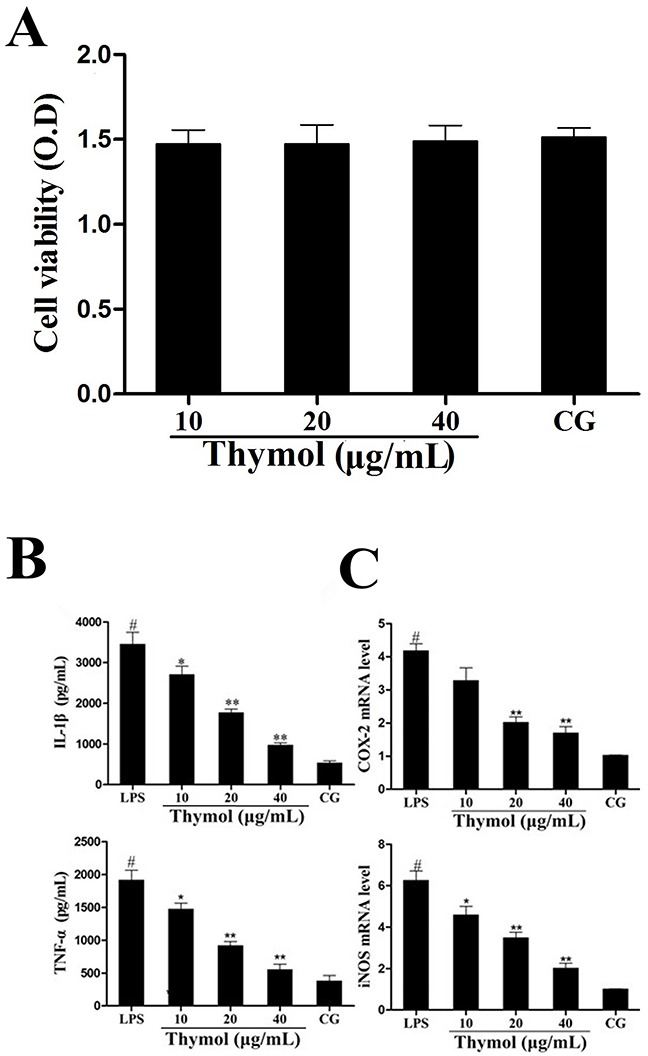
A. Effect of thymol on the cell viability of RAW264.7 cells **B**. Effects of thymol on the expression of TNF-α and IL-1β in LPS-stimulated RAW264.7 cells. **C**. Expression of iNOS and COX-2 mRNA in LPS-stimulated RAW264.7 cells. *GAPDH* served as the control. CG indicates the control group. LPS indicates the LPS-stimulated group. Data represent the mean ± S.E.M. of three independent experiments. ^#^p<0.01 *vs*. Control group. *p<0.05 *vs*. LPS group, **p<0.01 *vs*. LPS group.

The potential cytotoxicity of thymol on RAW264.7 cells was determined using the MTT assay. These results showed that cell viability was not affected by thymol administration (Figure 6A).

### Effect of thymol on cytokines levels

The expression of the cytokines TNF-α and IL-1β in RAW264.7 cells was determined using ELISA. These results indicated that the expression of TNF-α and IL-1β was significantly increased in the LPS group. However, treatment with thymol dose-dependently decreased the levels of TNF-α and IL-1β (Figure 6B). Moreover, iNOS and COX-2 are often used as inflammatory markers. We detected the expression of the cytokines iNOS and COX-2 in RAW264.7 cells using a qPCR assay. As shown in Figure 6C, LPS treatment significantly increased the expression of iNOS and COX-2. However, the expression of iNOS and COX-2 were dose-dependently inhibited by pretreatment with thymol.

### Effect of thymol on ROS production

**Figure 7:**
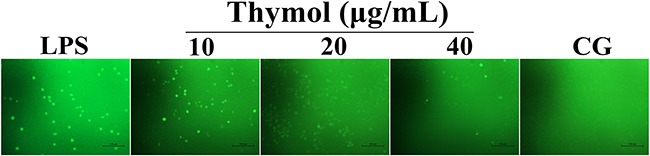
Effect of thymol on ROS production in LPS-stimulated RAW264.7 cells Microphotographs of ROS levels in LPS-stimulated RAW264.7 cells were obtained using fluorescence microscopy after DCF-DA staining (400× magnification, scale bar = 50 μm).

ROS, as signaling molecules involved in various processes, play a pivotal role in the inflammatory regulation [25]. Thus, we measured the generation of ROS to evaluate the antioxidant effect of thymol. As shown in Figure 7, these results showed that thymol dose-dependently suppressed LPS-induced ROS production in RAW264.7 cells.

### Effects of thymol on TLR4-mediated NF-κB pathway activation

**Figure 8:**
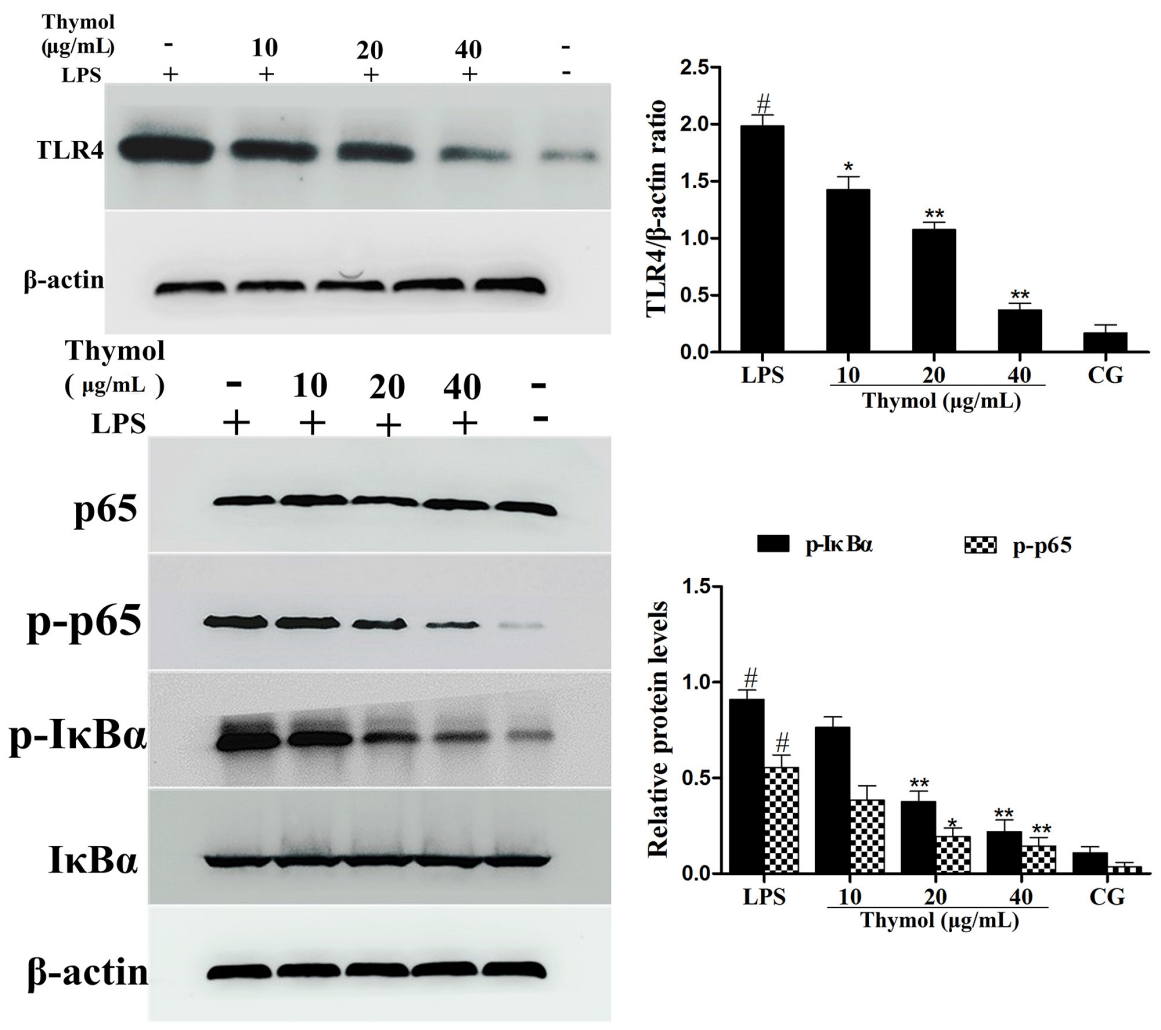
Effects of thymol on TLR4-mediated NF-κB pathway activation **A**. Expression of TLR4 in LPS-stimulated RAW264.7 cells. **B**. Expression of IκBα and p65 protein in LPS-stimulated RAW264.7 cells. Phosphorylation of IκBα and p65 were analyzed using phospho-specific antibodies. β-actin served as an internal control. CG indicates the control group. LPS indicates the LPS-stimulated group. Data represent the mean ± S.E.M. of three independent experiments. ^#^p<0.01 *vs*. Control group. *p<0.05 *vs*. LPS group, **p<0.01 *vs*. LPS group.

Since TLR4 plays a critical role in the regulation of the LPS-induced inflammatory pathway, the expression of TLR4 in LPS-stimulated RAW264.7 cells was detected by western blot. These results suggested that the expression of TLR4 was remarkably increased after being treated with LPS, which was dose-dependently down-regulated by thymol (Figure 8A).

It is well known that NF-κB participates in the regulation of inflammation. To examine the anti-inflammatory molecular mechanism of thymol, activation of the NF-κB pathway was assessed. As shown in Figure 8B, compared with the LPS group, the phosphorylation of the p65 and IκBα proteins was inhibited by thymol administration.

### Effect of thymol on the TLR4-dependent or TLR4-independent pathways

**Figure 9:**
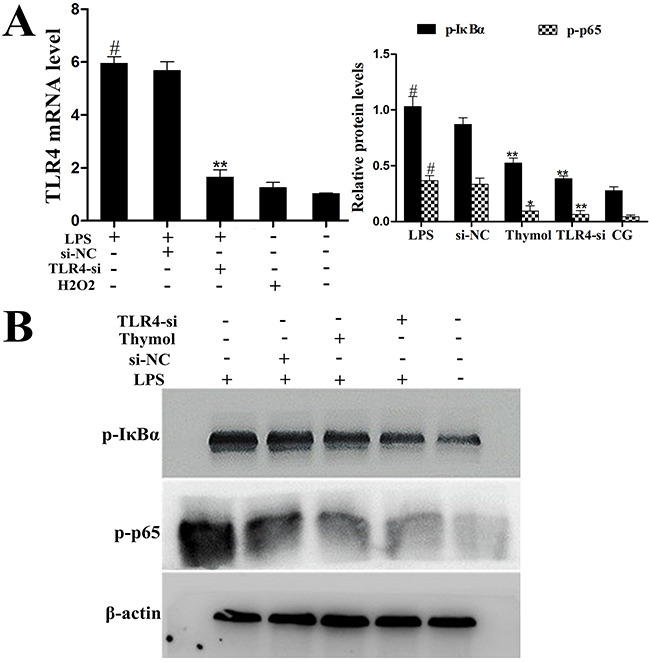
Analysis of TLR4-mediated NF-κB pathway expression in LPS-stimulated RAW264.7 cells **A**. The interfering efficiency of TLR4 siRNA was estimated using qPCR. *GAPDH* served as a control. **B**. Phosphorylation levels of p65 and IκBα were examined by western blotting after silencing of TLR4 using siRNA or thymol treatment in LPS-stimulated RAW264.7 cells. CG indicates the control group. LPS indicates the LPS-stimulated group. TLR4-si indicates the TLR4 siRNA group. si-NC indicates the siRNA-negative control group. Data represent the mean ± S.E.M. of three independent experiments. ^#^p<0.01 *vs*. Control group. *p<0.05 *vs*. LPS group, **p<0.01 *vs*. LPS group.

**Figure 10:**
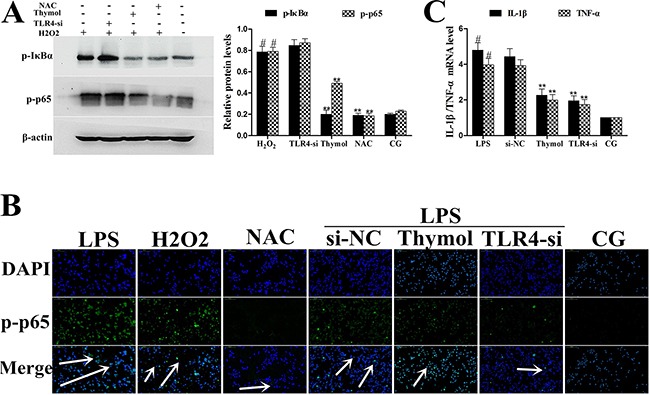
A. Effects of H_2_O_2_ on NF-κB activation after silencing of TLR4 in RAW264.7 cells Phosphorylation levels of p65 and IκBα were determined by western blotting after knockdown of TLR4 using siRNA or thymol, NAC pre-treatment. β-actin serves as an internal control. **B**. Translocation of NF-κB p65 in TLR4-si RAW264.7 cells challenged with LPS as assessed using immunofluorescence. **C**. Expression of the cytokines, IL-1β and TNF-α, was detected using qPCR. *GAPDH* served as a control. CG indicates the control group. LPS indicates the LPS-stimulated group. TLR4-si indicates the TLR4 siRNA group. NAC indicates the *N*-acetyl cysteine group. The white arrow indicates the translocation of p65. Data represent the mean ± S.E.M. of three independent experiments. ^#^p<0.01 *vs*. Control group. *p<0.05 *vs*. LPS group, **p<0.01 *vs*. LPS group.

To further confirm whether the effect of thymol on the NF-κB pathway is TLR4-dependent or TLR4-independent, specific interference RNA (TLR4-si) was used to silence TLR4 expression. When TLR4 was silenced, phosphorylation of NF-κB, p65 and IκBα were detected in RAW264.7 cells that had been treated with LPS or H_2_O_2_. These results showed that LPS induced excessive expression of TLR4, which was subsequently reduced by TLR4-si, and the LPS-induced phosphorylation of p65 and IκBα was also decreased by TLR4-si and thymol (40 μg/mL) (Figure 9A, 9B). ROS are involved in the regulation of NF-κB pathway activation and therefore elicit a wide spectrum of responses [26]. H_2_O_2_ is a strong oxidant and is often used as a representative ROS in modeling and inducing oxidative stress [27]. Thus, we also determined the effect of ROS on NF-κB activation in H_2_O_2_-stimulated, TLR4-si treated RAW264.7 cells by western blot. Interestingly, these results suggested that H_2_O_2_ treatment increased the phosphorylation of p65 and IκBα; however, phosphorylation of these proteins was decreased by pre-treatment with NCA or thymol (40 μg/mL) (Figure 10A). Moreover, further studies were performed to determine the translocation of NF-κB p65 in TLR4-si RAW264.7 cells that had been challenged with LPS using immunofluorescence assays (as shown in Figure 10B). In addition, LPS increased the expression of NF-κB downstream cytokines, IL-1β and TNF-α, which was dose-dependently alleviated by thymol (Figure 10C). The above results indicate that thymol inhibits NF-κB pathway activation in a TLR4-dependent or TLR4-independent manner.

## DISCUSSION

ROS play essential roles in protection from the invasion of microbial pathogens and are thought to be responsible for tissue damage, inflammation and cell signaling pathways [28, 29]. A recent study suggested that neutralization of ROS or inhibition of the redox pathway could relieve inflammation [30]. LPS, a bacterial cell wall component, is known to induce the production of several inflammatory cytokines, tissue edema, and injury [31]. As one of the most important immune cells, macrophage activation is a hallmark of inflammation and produces ROS, which trigger signal transduction pathways [32]. Although there have been many reports that have extensively described the anti-inflammatory function of thymol, a Chinese herbal medicine [19, 33], a detailed analysis of its molecular targets has not been completed until now. In the present study, an LPS-induced endometritis mouse model was first used to evaluate the anti-inflammatory effects of thymol *in vivo*, and its complex mechanism was uncovered in LPS-stimulated macrophages *in vitro*.

Thus far, researchers have paid more attention to traditional Chinese medicine due to its ability to lower toxicity and its increased efficacy in the treatment of inflammatory diseases [6, 34]. The results of *in vivo* experiments have indicated that thymol alleviates inflammatory injury and decreases MPO activity in LPS-induced mouse endometritis.

Inflammation is beneficial for the host defense against infection; however, excessive inflammatory response leads to injury [35]. Secretion of the cytokines IL-1β and TNF-α increases dramatically during the process of inflammatory pathological development [6, 24]. IL-1β, the master regulator of inflammation, is well known to induce inflammation by activating a cascade of molecular pathways [36]. In addition, TNF-α, iNOS and COX-2 were also markedly increased in LPS-stimulated inflammatory disease [34]. *In vitro* studies revealed that thymol inhibited the expression of TNF-α, IL-1β, iNOS and COX-2 in LPS-stimulated RAW264.7 cells. Moreover, some reports have indicated that these pro-inflammatory cytokines are mainly activated by the NF-κB signaling pathway, which is also mediated by ROS [12, 25]. NF-κB, a nuclear transcription factor, is a regulator of inflammatory processes [37]. Upon stimulation, NF-κB moves to the nucleus and induces the expression of various genes, including IκBα [38]. In the present study, thymol administration inhibited the phosphorylation of IκBα and p65 in LPS-induced mouse endometritis and RAW264.7 cells.

TLR4, a Type I transmembrane receptor, is activated by LPS and plays an integral role in the innate immune system [6]. It is well known that LPS activates the TLR4-mediated signaling pathway and leads to the activation of NF-κB. Previous studies have reported that the activation of NF-κB is regulated by ROS in LPS-induced inflammation [39]. Therefore, we sought to investigate whether the anti-inflammatory mechanism of thymol inhibited the activation of NF-κB via TLR4-independent or TLR4-dependent pathways. In the present study, we found that LPS increased the expression of TLR4, which was reduced by thymol treatment. When TLR4 was silenced, phosphorylation of p65 and IκBα were decreased by TLR4-si and thymol (40 μg/mL) in LPS-induced RAW264.7 cells. H_2_O_2_ is a strong oxidant and induces excessive ROS production; therefore, it contributes to signaling cascades, such as the NF-κB pathway [40]. Moreover, *N*-acetyl cysteine (NAC) is an efficient antioxidant and is often used as a positive control [32]. We observed that phosphorylation of p65 and IκBα was greatly increased in the H_2_O_2_ treatment group, and phosphorylation of these proteins was decreased by NCA or thymol (40 μg/mL). Taken together, these results indicate that thymol inhibited the activation of NF-κB via TLR4-mediated or ROS-regulated signaling pathways.

In conclusion, the current study clearly demonstrates that thymol can effectively inhibit the expression of pro-inflammatory cytokines in LPS-induced mouse endometritis. The promising anti-inflammatory effect of thymol on LPS-stimulated RAW264.7 cell is relevant to TLR4-mediated or ROS-dependent NF-κB signaling pathways, thereby suppressing LPS-induced inflammatory responses.

## MATERIALS AND METHODS

### Reagents

Thymol [high-performance liquid chromatography (HPLC) purity ≥ 98%] was purchased from Shanghai Yuanye Bio-Technology Co., Ltd. (Shanghai, China). The purity of thymol was determined by HPLC. This assay was performed on EChrom2000 DAD Data System (Elite, China). Chromatography was performed using a Hyper ODS-2 C18 column (5 μm, 250×4.6 mm, Dikma Technology, California, USA). Elution was performed with acetonitrile/water (20: 80), and the flow rate was 1.0 mL/min with DAD detection at 268 nm (Figure 1B). *N*-acetyl cysteine (NAC) was purchased from the Beyotime Institute of Biotechnology (Shanghai, China). LPS (*E. coli* 055:B5) was purchased from Sigma Chemical CO (St. Louis, USA). Primary antibodies for β-actin and NF-κB were purchased from Cell Signaling Technology (Beverly, USA). Other antibodies were obtained from Santa Cruz Biotechnology (Dallas, USA).

### Animal treatment and experimental groups

A total of 50 female BALB/c mice (8 weeks old, approximately 25 g in weight) were used in this study. The mice were obtained from Hubei Provincial Center for Experimental Animal Research (Wuhan, China). The mice were fed a standard diet and housed in a temperature-controlled room with a 12 h dark/light cycle for one week prior to the experiments. The present study was performed according to the guidelines of the Huazhong Agricultural University Animal Care Committee and the care and use of Laboratory Animals published by the US National Institutes of Health.

Mice were randomly divided into the following 5 groups with 10 mice in each group to establish the model of endometritis: a control group, thymol (10, 20, and 40 mg/kg) + LPS groups, and an LPS group. Thymol was solubilized by dimethyl sulfoxide (the DMSO concentration in the solution was not greater than 0.1%) to obtain final concentrations of 10, 20, and 40 mg/kg. The method for inducing the endometritis model in mice was performed as previously described [34]. Briefly, each side of the mouse uterus was perfused with 50 μL of LPS (1 mg/mL) to induce endometritis. After 24 h, the blank group received normal saline. The thymol groups received an intraperitoneal injection of differing concentrations of thymol (10, 20, and 40 mg/kg) three times (once every six hours). Next, the mice were sacrificed by CO_2_ inhalation, and the uterus tissues were collected and stored at -80°C.

### Histopathological assessment

Uterus tissues were isolated, and approximately one centimeter-sized tissue was fixed in10% formalin for subsequent histopathological experiments. The tissues were dehydrated, paraffin embedded, and then cut into 5-μm-thick sections for hematoxylin and eosin (H&E) staining. Finally, the sections were observed using an optical microscope (Olympus, Japan).

### Myeloperoxidase (MPO) analysis

The MPO activity was measured according to the manufacturer's instructions. Briefly, uterine tissue, weighing approximately 100 mg, was fixed with phosphate buffered saline (PBS, weight/volume ratio 1:19) and homogenized. The supernatants were analyzed using the MPO kit (Jiancheng biotechnology, China) and detected at an absorbance value of 460 nm.

### Cell culture and treatment

RAW264.7 cells were purchased from the American Type Culture Collection (Manassas, USA) and cultured in DMEM with 10% fetal bovine serum at 37°C with 5% CO_2_. The cells were pretreated with different concentrations of thymol (10, 20, and 40 μg/mL) for 1 h and then stimulated with LPS (1 μg/mL) for 3 h.

### Cell viability assay

The cytotoxic effects of thymol on RAW264.7 cells were examined with the 3-[4,5-dimethylthiazol-2-yl]-2,5 diphenyl tetrazolium bromide (MTT) assay. The cells (1×10^4^ cells mL^−1^) were seeded onto 96-well plates at 37°C for 6 h. Next, the cells were treated with different concentrations of thymol (10, 20, and 40 μg/mL) for 24 h. Twenty μL of MTT (5 mg/mL) was added for 4 h, the supernatant was discarded, and 100 μL of DMSO was added per well. The optical density (OD) was read at an absorbance value of 570 nm using a microplate reader (Thermo, USA).

### Cytokine analysis

The uterine tissues were homogenized in pre-chilled PBS, centrifuged and the supernatants were collected. RAW264.7 cells were seeded onto a 6-well-plate, and the cells were treated as indicated. The tissue and cell supernatants were harvested to detect the expression of IL-1β and TNF-α using ELISA kits (Bio-Swamp, China) according to the manufacturer's instructions. The absorbance value was read at 450 nm using a microplate reader (Thermo, USA).

### Determination of ROS in RAW264.7 cells

ROS production in RAW264.7 cells was measured using the oxidative conversion of cell permeable 2′, 7′-dichlorofluorescein diacetate (DCFH-DA, Beyotime, China) to fluorescent dichlorofluorescein (DCF). Cells were seeded at a density of 1×10^5^ cells mL^−1^ into 6-well plates. Next, they were incubated with control media or 1 μg/mL LPS in the presence or absence of thymol (10, 20, and 40 μg/mL) for 3 h. The cells were incubated with DCFH-DA for 30 min at 37°C and then washed three times with PBS to remove extracellular DCFH-DA. The DCF fluorescence was observed using a fluorescence microscope (Leica, Germany).

### Quantitative PCR assay

Total RNA was extracted from RAW264.7 cells with TRIzol (Invitrogen, USA). Next, cDNA was synthesized using a reverse transcription kit (Takara, Japan) and primers, which are shown in Table 1. The PCR reaction (40 cycles) was performed using a SYBR qPCR Mix (Roche, Swiss). The housekeeping gene GAPDH served as an internal standard. The relative quantification of the target gene expression levels was calculated using the 2^−ΔΔCt^ comparative approach.

**Table 1:** Primers used for qPCR

Name	Sequence (5′→3′):Forward and reverse	GenBankAccession No.	Product Size(bp)
TLR4	TTCAGAGCCGTTGGTGTATCCTCCCATTCCAGGTAGGTGT	NM_021297.2	170
TNF-α	CTTCTCATTCCTGCTTGTGACTTGGTGGTTTGCTACG	NM_013693.3	198
IL-1β	CCTGGGCTGTCCTGATGAGAGTCCACGGGAAAGACACAGGTA	NM_008361.4	131
COX-2	GAAGTCTTTGGTCTGGTGCCTGGTCTGCTGGTTTGGAATAGTTGC	NM_011198.4	133
iNOS	GGAGCGAGTTGTGGATTGTCGTGAGGGCTTGGCTGAGTGAG	NM_001313922.1	123
GAPDH	CAATGTGTCCGTCGTGGATCT1GTCCTCAGTGTAGCCCAAGATG	NM_001289726.1	124

### Western blotting analysis

The total proteins of the uterine tissue and RAW264.7 cells were extracted with a RIPA lysis solution containing a phosphatase repressor. The concentration of proteins was determined using a BCA kit. Next, samples with equal amounts of protein were fractionated on a 10% SDS polyacrylamide gel and then transferred onto a polyvinylidene difluoride membrane. The membrane was incubated in blocking buffer (5% skim milk) and subsequently treated with primary antibody (1:1000 dilution) at 4°C overnight and then washed three times with PBS for 30 min. Next, the membranes were incubated with secondary antibody for 1 h at room temperature, and the protein expression levels were determined using the ECLPlus western blot Detection System. β-actin served as an internal standard.

### TLR4-*siRNA* transfection

Negative control siRNA mice and TLR4-siRNA mice were obtained from Ribo (Guangzhou, China). For TLR4-*siRNA* transfection, RAW264.7 cells were seeded onto 6-well plates and allowed to reach approximately 70% confluency. Transfection was performed in Opti-MEM with NC-*siRNA* or TLR4-*siRNA* using Lipofectamine™ 2000 (Invitrogen, USA) for 6 h, and the medium was replaced with fresh RPMI-1640. Next, the treatment group was pre-treated with thymol (40 μg/mL) for 1 h, and then LPS (1 μg/mL) was added for 3 h. For the LPS group, cells were treated with LPS for 3 h. H_2_O_2_ (1 mM) or *N*-acetyl cysteine (NAC, 15 mM) was considered the negative or positive ROS control, respectively, in order to compare similar processes as described in a previous study [41], and the cells were cleaved for further analysis.

### Immunofluorescence staining

Uterine tissues were fixed in 10% buffered formaldehyde for 24 h and then embedded in paraffin. Tissue sections were permeabilized with PBS containing 0.3% Triton X-100 (Sigma, USA) and 10% BSA. RAW264.7 cells (1×10^5^ cells mL^−1^) were seeded onto a twelve-well-plate. After the cells were treated as indicated, immunofluorescence staining was performed. Sections of tissue and cells were incubated with special antibody for p-p65 (1:100) overnight at 4°C and then incubated with a Cy3 secondary antibody (1:200) in the dark for 2 h at 25°C. Next, p-p65 protein was mounted using a mounting medium supplemented with 4,6-diamidino-2-phenylindole (DAPI, Beyotime, China) for nuclear counterstaining and observed using fluorescence microscopy (Olympus, Japan).

### Statistical analyses

Statistical data were represented as the mean ± S.E.M. Data were assessed using Student's *t*-test or one-way analysis of variance (ANOVA). *p-value* ≤ 0.05 were considered a statistically significant difference.
